# Dynamics of Body Mass Index and Serum Albumin Levels Before and After the Intensive Phase of Tuberculosis Treatment

**DOI:** 10.12688/f1000research.179357.1

**Published:** 2026-04-19

**Authors:** Lisna Rosalia Agaus, Muh. Ilyas, Agus Sudarso, Syakib Bakri, Haerani Rasyid, Andi Alfian Zainuddin

**Affiliations:** 1 Faculty of Medicine, Hasanuddin University, Makassar, South Sulawesi, Indonesia; 2 Department of Public Health and Community Medicine, Hasanuddin University, Makassar, South Sulawesi, Indonesia

**Keywords:** Tuberculosis, Body Mass Index, Serum Albumin, Nutritional Status, Intensive Phase Treatment

## Abstract

**Introduction:**

Tuberculosis remains a major global health problem and is frequently associated with malnutrition, which can negatively affect treatment outcomes. This study aimed to evaluate changes in body mass index (BMI) and serum albumin levels before and after the intensive phase of tuberculosis treatment.

**Methods:**

An observational analytic study with a prospective cohort design was conducted at Dr. Wahidin Sudirohusodo Hospital, Makassar. A total of 175 newly diagnosed tuberculosis patients were enrolled. BMI and serum albumin levels were measured at baseline and after completion of the two-month intensive treatment phase. Paired sample *t*-tests were used to assess differences between pre- and post-treatment values.

**Results:**

BMI increased significantly from 19.21 ± 3.55 kg/m ^2^ to 19.78 ± 3.50 kg/m ^2^ ( *p* < 0.0001), corresponding to a 2.96% improvement. Serum albumin levels also increased significantly from 3.06 ± 0.62 g/dL to 3.25 ± 0.64 g/dL ( *p* < 0.0001), with an average improvement of 6.17%. Additionally, the proportion of patients with normal nutritional status increased, while the proportion of underweight patients decreased significantly after treatment ( *p* = 0.002).

**Conclusions:**

Intensive-phase anti-tuberculosis therapy is associated with significant improvements in both anthropometric and biochemical nutritional parameters. Routine monitoring of BMI and serum albumin may serve as practical indicators for assessing treatment response and guiding nutritional interventions in tuberculosis patients.

## Introduction

Tuberculosis (TB) remains a major global public health problem and one of the leading causes of death from infectious diseases. Caused by *Mycobacterium tuberculosis*, TB primarily affects the lungs but may involve multiple organs. ^1, 2^ Despite the availability of effective treatment, TB continues to contribute substantially to global morbidity and mortality. ^3^ In 2022, TB ranked as the second leading cause of death from a single infectious agent after COVID-19 and caused nearly twice as many deaths as HIV/AIDS, with approximately 7.5 million newly diagnosed cases reported worldwide, the highest number since global TB surveillance began. ^4, 5^

Indonesia remains among the countries with the highest TB burden globally, ranking second after India in total cases. ^4, 6^ In 2022, South Sulawesi Province reported 24,209 TB cases, with an estimated incidence of 35,210 cases and a treatment coverage rate of 69%. ^7^ These findings underscore the persistent challenge of TB control in Indonesia and the need for improved clinical management and monitoring. ^8^

Malnutrition is a common comorbidity in TB and plays a critical role in disease progression and treatment outcomes. Undernutrition in TB patients results from anorexia, increased metabolic demands, impaired nutrient absorption, and enhanced protein catabolism. ^9, 10^ The relationship between TB and malnutrition is bidirectional, as TB worsens nutritional status while malnutrition impairs immune function and treatment effectiveness. ^11^

Nutritional status in TB patients is commonly assessed using anthropometric and biochemical parameters. Body mass index (BMI) is a simple and widely used indicator of nutritional status, while serum albumin reflects both nutritional reserves and systemic inflammatory activity. ^12, 13^ Reduced BMI and hypoalbuminemia are frequently observed in TB patients and are associated with poor clinical outcomes, delayed recovery, and increased complications. ^14^ Improvements in BMI and serum albumin have been linked to better treatment responses in TB. ^12, 15, 16^

Standard TB treatment consists of an intensive phase followed by a continuation phase. The intensive phase, typically lasting two months, aims to rapidly reduce mycobacterial load, alleviate symptoms, and prevent drug resistance. ^17^ Although this phase is expected to improve nutritional status, adverse drug effects such as gastrointestinal symptoms and hepatotoxicity may hinder nutritional recovery. ^18, 19^

Given the close interaction between TB and nutritional status, monitoring changes in BMI and serum albumin during treatment is essential. However, data on the dynamics of these parameters before and after the intensive phase of TB therapy remain limited, particularly in Indonesia. Therefore, this study aims to analyze changes in BMI and serum albumin levels before and after the intensive phase of tuberculosis treatment to better characterize nutritional responses and support comprehensive patient management.

## Methods

### Study design and setting

This study was an observational analytic study with a prospective cohort (longitudinal) design using a pre–post approach. The research was conducted at Dr. Wahidin Sudirohusodo Hospital, Makassar, and its affiliated health facilities from October 2024 until the required sample size was achieved. Data were collected primarily through direct measurements and laboratory examinations on newly diagnosed tuberculosis (TB) patients who had just initiated anti-tuberculosis treatment.

### Study population and sampling

The target population consisted of all patients aged ≥18 years who were newly diagnosed with tuberculosis and started TB treatment at Dr. Wahidin Sudirohusodo Hospital and its network. Subjects were recruited using a consecutive sampling technique, in which all patients fulfilling the eligibility criteria during the study period were included until the minimum sample size was met.

### Eligibility criteria

Participants included in this study were patients aged ≥18 years with newly diagnosed tuberculosis, either bacteriologically confirmed or clinically diagnosed. All subjects were individuals who were initiating anti-tuberculosis treatment for the first time and were willing to participate in the study by providing written informed consent.

Patients were excluded if they had comorbid HIV/AIDS, a history of chronic liver disease, or chronic kidney disease. In addition, patients currently receiving medications that could affect nutritional status, such as corticosteroids, anticancer agents, or antiepileptic drugs, were also excluded from participation.

During the course of the study, subjects were considered drop-outs if they experienced adverse drug reactions or complications during the intensive phase of tuberculosis treatment, demonstrated poor adherence to therapy or were lost to follow-up, or developed other medical conditions that could potentially influence nutritional status during the study period.

### Data collection procedures

Data collection was conducted through a series of structured procedures beginning with the screening of all suspected tuberculosis patients in both outpatient and inpatient units. Patients who met the predetermined inclusion and exclusion criteria were selected as potential study subjects. Each eligible patient received a detailed explanation regarding the objectives and procedures of the study, after which written informed consent was obtained. Baseline demographic data were then collected, followed by measurement of body weight and height to calculate Body Mass Index (BMI). A complete physical examination and assessment of vital signs were also performed. Baseline laboratory data were retrieved, including serum albumin levels, tuberculosis microbiological test results (smear, culture, and TCM), and HIV status. After the completion of two weeks of intensive phase anti-tuberculosis therapy, participants underwent reassessment, which included repeat measurements of body weight, height, BMI, and serum albumin levels. All collected data were subsequently processed and analyzed using appropriate statistical methods.

### Data analysis

All collected data were analyzed using Statistical Package for Social Sciences (SPSS) version 22. Descriptive statistics were used to summarize subject characteristics. The comparison between pre- and post-treatment measurements was analyzed using the Paired Sample T-Test. A p-value <0.05 was considered statistically significant.

## Results

### Characteristics of study subjects

This study was conducted from October 2024 to September 2025 at Dr. Wahidin Sudirohusodo Hospital, Makassar, and its affiliated hospitals. A total of 175 newly diagnosed tuberculosis patients who had initiated anti-tuberculosis treatment were included, comprising both inpatient and outpatient cases. As shown in Table 1, most patients were aged 18–60 years (66.29%), with 33.71% aged over 60 years. Male patients predominated (66.86%). Based on Molecular Rapid Test (TCM) results, 58.29% had bacteriologically confirmed tuberculosis, while 41.71% were clinically diagnosed. The mean body weight was 49.33 ± 10.06 kg, with a mean BMI of 19.21 ± 3.55 kg/m ^2^. Nearly half of the patients had normal BMI (46.86%), while a substantial proportion were underweight (41.14%). Smaller proportions were overweight or obese. The mean serum albumin level was 3.06 ± 0.62 g/dL, indicating generally suboptimal nutritional status. Overall, the study population was predominantly male, of productive age, with a high proportion of bacteriologically confirmed TB and a tendency toward poor nutritional status during the intensive treatment phase.

**Table 1.** Demographic characteristics of study subjects.

Variable	n	%
Age		
18–60 years	116	66.29
> 60 years	59	33.71
Gender		
Male	117	66.86
Female	58	33.14
TCM Result		
Bacteriologically Confirmed TB	102	58.29
Clinical TB	73	41.71
Nutritional Status		
Underweight	72	41.14
Normal	82	46.86
Overweight	11	6.29
Obesity I	9	5.14
Obesity II	1	0.57
Albumin Level		
Low	126	72
Normal	49	28

Changes in Body Mass Index and Serum Albumin Levels in Patients During the Intensive Phase of TB Treatment.

**Figure 1.**
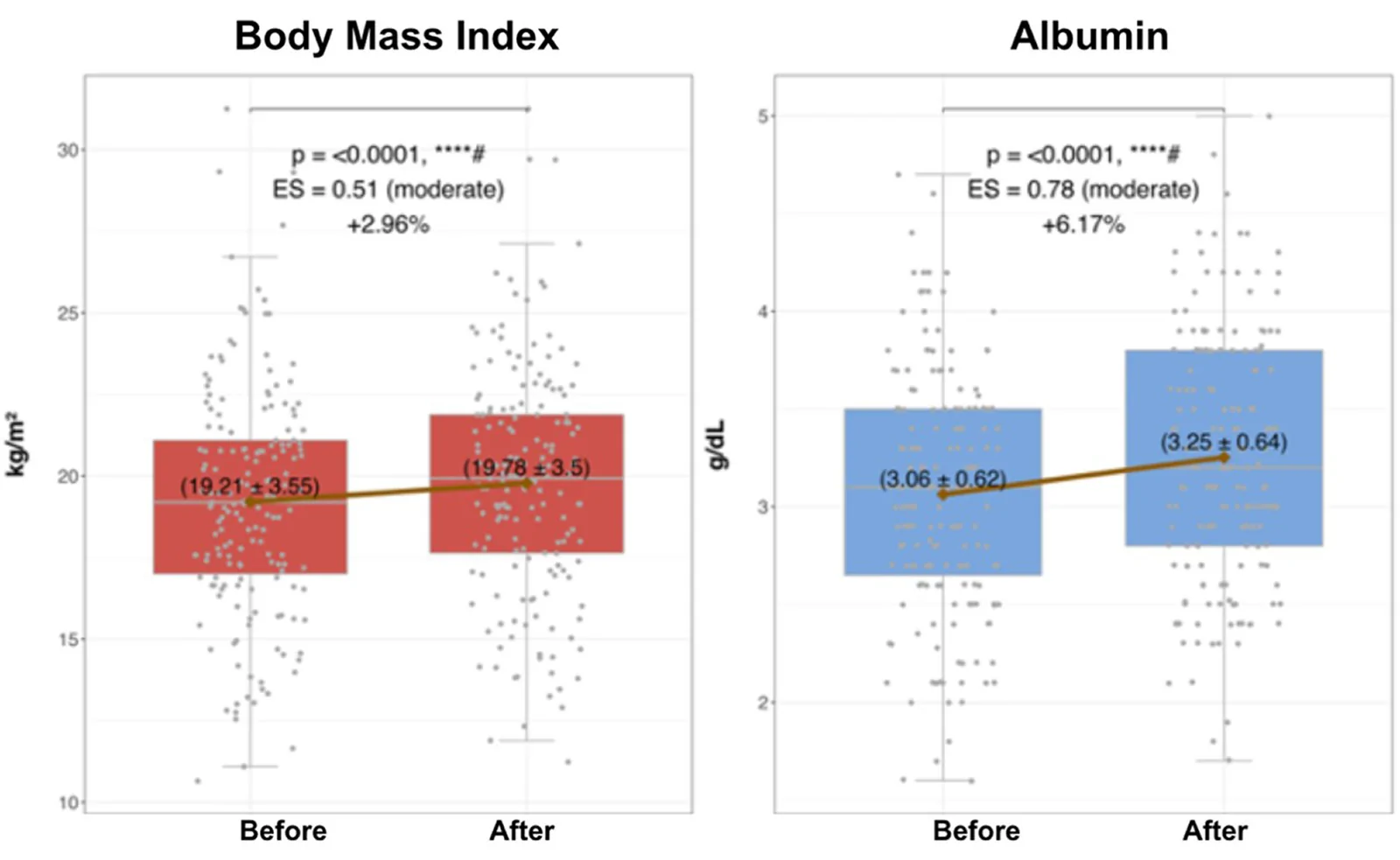
Diagram of changes in bmi and serum albumin levels in patients during the intensive phase of TB treatment. Comparison test with Stuart Maxwell Test. Significant codes: <0.0001′****’ < 0.001′***’ < 0.01′**’ < 0.05′*’ not significant ‘ns’.

In this study, an analysis was conducted to evaluate changes in BMI and serum albumin levels in patients undergoing the intensive phase of tuberculosis treatment, both before and after therapy, using the paired T-test. The results of the analysis demonstrated significant changes in both BMI and serum albumin levels among patients during the intensive phase of TB treatment. The findings of this analysis are presented in Table 2 and Figure 1. BMI increased from 19.21 ± 3.55 to 19.78 ± 3.50 after the intensive phase of treatment. This increase in BMI (Δ 0.57 kg/m ^2^), equivalent to a 2.96% improvement, was statistically significant (p < 0.0001). Similarly, serum albumin levels showed an increase from 3.06 ± 0.62 g/dL to 3.25 ± 0.64 g/dL following the intensive phase of therapy. The increase in albumin level (Δ 0.19 g/dL), representing a 6.17% improvement, was also found to be statistically significant (p < 0.0001). Overall, intensive phase treatment provides a significant positive impact on both Body Mass Index and serum albumin levels.

**Table 2.** Changes in body mass index and serum albumin levels in patients during the intensive phase of TB treatment.

Variable	Intensive phase (Mean ± SD)		Change Δ (% increase) after	p-value
	Before	After		
BMI	19.21 ± 3.55	19.78 ± 3.50	0.57 (2.96%)	<0.0001
Albumin (g/dL)	3.06 ± 0.62	3.25 ± 0.64	0.19 (6.17%)	<0.0001

Comparison test: Paired T-test. Significance codes: <0.0001 (), <0.001 (), <0.01 (**), <0.05 (), not significant (ns)

### Changes in nutritional status before and after the intensive phase of TB treatment

**Figure 2.**
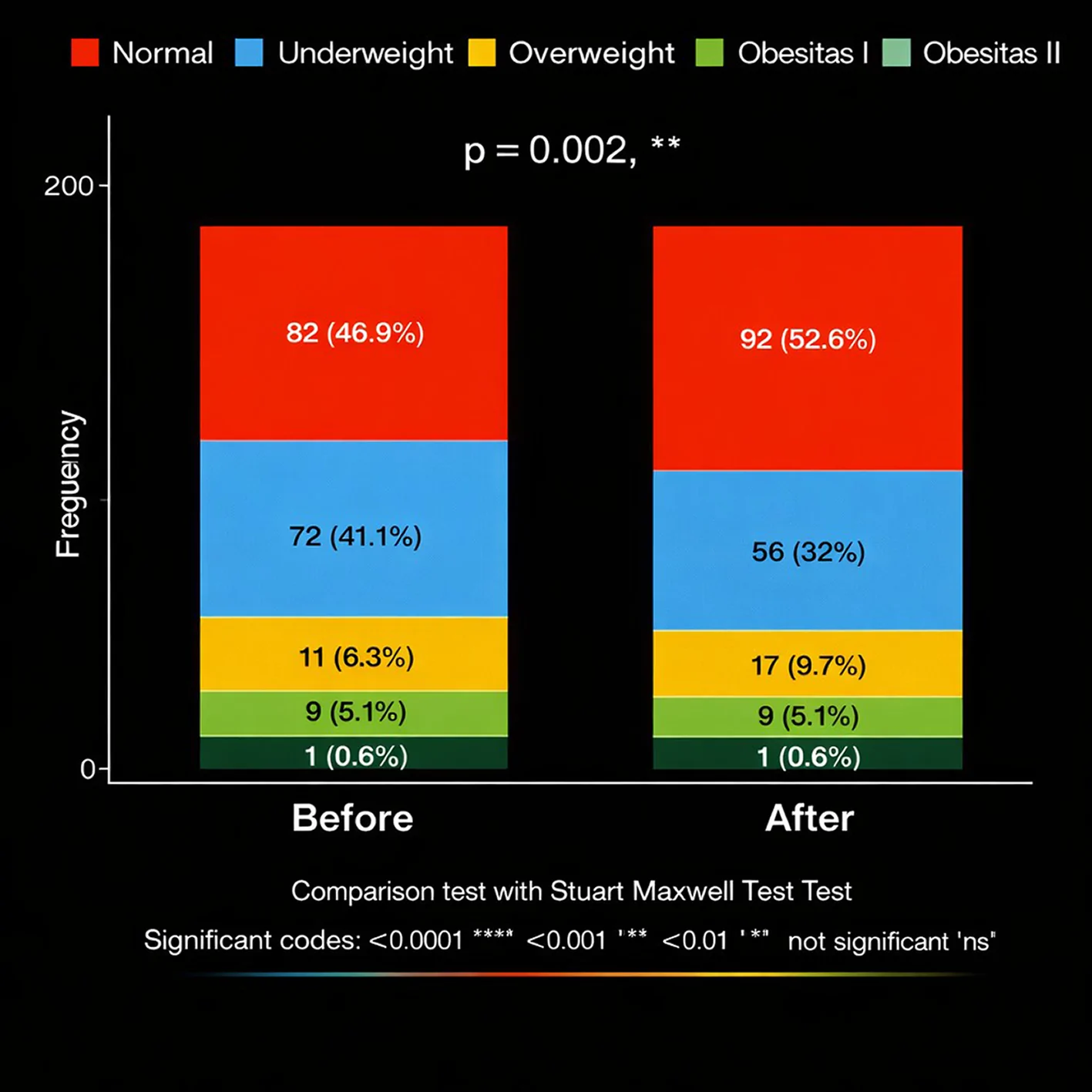
Graph of changes in nutritional status in patients before and after the intensive phase of TB treatment.

In this study, changes in the nutritional status of patients before and after the intensive phase of tuberculosis treatment were analyzed based on BMI measurements. The distribution of nutritional status categories and their changes are presented in Table 3 and illustrated in Figure 2. The analysis of nutritional status changes demonstrated a statistically significant shift in distribution between the pre- and post-intensive treatment phases, with a p-value of 0.002 based on the Stuart–Maxwell Test. These findings indicate a meaningful improvement in nutritional condition following the intensive phase of therapy. Prior to treatment, the majority of patients were classified as having normal nutritional status (46.9%), followed by those categorized as underweight (41.1%). The remaining patients were distributed across overweight (6.3%), obesity class I (5.1%), and obesity class II (0.6%).

**Table 3.** Changes in nutritional status and serum albumin levels before and after the intensive phase of TB treatment.

Variable		Before therapy (%)	After therapy (%)
Nutritional Status	Underweight	72 (41.14%)	56 (32%)
	Normal	82 (46.86%)	92 (52.6%)
	Overweight	11 (6.29%)	17 (9.7%)
	Obesity I	9 (5.14%)	9 (5.14%)
	Obesity II	1 (0.57%)	1 (0.57%)
Albumin Level	Low	126 (72%)	110 (62.86%)
	Normal	49 (28%)	65 (37.14%)

After completion of the intensive phase of TB treatment, an increase in the proportion of patients with normal nutritional status was observed, rising to 52.6%, while the proportion of underweight patients decreased to 32%. In addition, the percentage of overweight patients increased from 6.3% to 9.7%. The proportions of patients in obesity class I and class II remained relatively stable, at 5.1% and 0.6%, respectively. These changes indicate an overall improvement in the nutritional status of patients following intensive TB treatment. The increase in the proportion of patients with normal BMI and the reduction in the underweight group suggest a positive trend toward weight recovery, likely resulting from improved metabolic conditions, reduction of inflammatory processes, and enhanced nutritional intake during treatment. Therefore, the intensive phase of TB therapy not only plays a crucial role in infection control but also contributes significantly to the improvement of patients’ nutritional status, which is essential for optimal recovery and better quality of life.

## Discussion

This study demonstrated significant nutritional improvement among tuberculosis patients after the intensive phase of treatment. Mean BMI increased from 19.21 to 19.78 (2.96%, p < 0.0001), consistent with previous reports. Mesele Wassie et al. (2014) and Kumar et al. (2025) similarly observed early BMI gains following intensive anti-tuberculosis therapy, reflecting effective bacterial suppression and clinical recovery. ^20, 21^

The increase in BMI can be explained by reduced systemic inflammation. Ko et al. (2020) described that declining mycobacterial load leads to lower pro-inflammatory cytokine activity, improving appetite and nutrient utilization. ^10^ Wagnew et al. (2024) further noted that nutritional recovery represents a shift from a hypercatabolic to a more stable metabolic state. ^22^ However, as shown by Kalva et al. (2023) and Sontakke et al. (2024), BMI responses vary depending on drug tolerance, baseline nutrition, and socioeconomic factors. ^23^

Serum albumin levels also increased significantly after treatment, from 3.06 ± 0.62 g/dL to 3.25 ± 0.64 g/dL (p < 0.0001). Feleke et al. (2019) reported that hypoalbuminemia in TB is driven by chronic inflammation and suppressed hepatic synthesis, while Hung et al. (2023) showed that effective therapy restores protein metabolism. ^9, 14^ Similar albumin improvements during the intensive phase were documented by Liu et al. (2020) and Ko et al. (2020), although Edyson et al. (2024) noted that hepatotoxicity may blunt biochemical recovery in some patients. ^10, 24, 25^

This study also found a parallel improvement in BMI and serum albumin. Simbolon and Lombo (2016) demonstrated a positive correlation between these parameters, supporting their use as complementary indicators of nutritional and inflammatory recovery. ^15^ In line with Chandrasekaran et al. (2017), a significant shift toward normal nutritional status was observed after treatment. ^26^

Nevertheless, some patients experienced nutritional deterioration during the intensive phase. Wulandari and Karolina (2023) and Maaz et al. (2024) attributed this to drug-related gastrointestinal effects and high medication burden, reinforcing the bidirectional relationship between tuberculosis and malnutrition described by Chandrasekaran et al. (2017). ^26– 28^ Clinically, routine monitoring of BMI and serum albumin is essential during intensive-phase TB treatment. As recommended by Wunderle et al. (2024), early identification of patients with poor nutritional response allows timely intervention and may improve treatment outcomes. ^29^

Limitations include the short follow-up limited to the intensive phase and the lack of detailed assessment of dietary intake, adherence, and socioeconomic factors. Future longitudinal studies are needed to clarify long-term nutritional trajectories. In conclusion, intensive-phase tuberculosis treatment is associated with significant improvements in BMI, serum albumin, and overall nutritional status, supporting their role as practical markers of clinical recovery.

## Conclusion

Intensive-phase tuberculosis treatment resulted in significant improvement in the nutritional status of patients. BMI increased from a mean of 19.21 ± 3.55 kg/m ^2^ to 19.78 ± 3.50 kg/m ^2^, representing an average improvement of 0.57 kg/m ^2^ (2.96%). Similarly, serum albumin levels rose from 3.06 ± 0.62 g/dL to 3.25 ± 0.64 g/dL, with a mean increase of 0.19 g/dL (6.17%). These findings indicate that effective anti-tuberculosis therapy during the intensive phase is associated with measurable recovery in both anthropometric and biochemical nutritional parameters. Monitoring BMI and serum albumin can therefore serve as practical and objective indicators for evaluating therapeutic response and nutritional improvement in tuberculosis patients.

## Ethical considerations

This study was conducted in accordance with ethical standards for medical research involving human participants. Ethical approval was obtained from the Health Research Ethics Committee of the Faculty of Medicine, Hasanuddin University, (Ethical Approval No. 175/UN4.6.4.5.31/PP36/2025; 14 March 2025), prior to data collection, and no separate hospital-level approval was required. All participants received a detailed explanation of the study objectives, procedures, potential benefits, and possible risks, and provided written informed consent before participation. Participation was voluntary, with the right to withdraw at any time without consequences for medical care, and the confidentiality and anonymity of all personal and clinical data were strictly maintained throughout data collection, analysis, and publication.

## Data Availability

The data supporting the findings of this study are available from the corresponding author upon reasonable request. Due to ethical restrictions and the protection of patient confidentiality, the dataset is not fully publicly available. However, a de-identified dataset and relevant materials can be accessed via Zenodo at: https://bit.ly/4ttsnIf. ^30^ Access to additional data may be granted upon reasonable request to the corresponding author, subject to approval and applicable data-sharing conditions.
