# Supplementary material for: Dynamics of Body Mass Index and Serum Albumin Levels Before and After the Intensive Phase of Tuberculosis Treatment
Source: F1000Res. 2026 Jul 23;15:594. Originally published 2026 Apr 19. [Version 2] doi: 10.12688/f1000research.179357.2 (PMC13550917; doi:10.12688/f1000research.179357.2)
Supplement: Peer review history

# Reviewer report: Reviewer response for version 1

**DOI:** 10.5256/f1000research.197862.r494262

**Published:** 2026-07-06

**Authors:** Falak Pahwa

**Affiliations:** 1 The University of Chicago, Illinois, Chicago, USA

Agaus et al demonstrate changes in BMI and serum albumin before and after the intensive phase of TB treatment in an Indonesian cohort. It is a meticulous study and following up on patients is a commendable task, and such data are valuable for assessing TB treatment response using simple biochemical and anthropometric assays.

 However, I have a few minor comments that should be addressed before publication:

1. Have the authors analysed changes stratified by sex and/or age as a parameter. It would be important to determine whether BMI and albumin changes are consistent across males versus females and among younger versus older patients.
2. The follow-up period appears limited to two weeks after the intensive phase. Do the authors have data on a subset of patients who continued through the full course of TB therapy? Inclusion of outcomes from patients who completed treatment, along with bacillary burden or conversion data, would strengthen the findings.
3. The authors suggest that improvements in BMI may be related to reduced systemic inflammation. If available, inclusion of data on pro-inflammatory cytokines would support this interpretation.
4. It is interesting to see that some of the patients became overweight. Could the authors clarify this observation? Additionally, please specify when (time of day) weight and other measurements were taken, and was it consistent for all the recruited patients.
5. Early improvements can give the impression of full recovery, but many patients remain undernourished relative to the general population even after treatment and still have elevated long‑term morbidity and mortality. Expanding this point in the discussion would improve the manuscript.

Is the work clearly and accurately presented and does it cite the current literature?

Partly

If applicable, is the statistical analysis and its interpretation appropriate?

Yes

Are all the source data underlying the results available to ensure full reproducibility?

Yes

Is the study design appropriate and is the work technically sound?

Partly

Are the conclusions drawn adequately supported by the results?

Partly

Are sufficient details of methods and analysis provided to allow replication by others?

Partly

Reviewer Expertise:

Immunology, TB

I confirm that I have read this submission and believe that I have an appropriate level of expertise to confirm that it is of an acceptable scientific standard.

# Reviewer report: Reviewer response for version 1

**DOI:** 10.5256/f1000research.197862.r478779

**Published:** 2026-05-07

**Authors:** Sri Jayanti

**Affiliations:** 1 Eijkman Research Center for Molecular Biology, National Research and Innovation Agency, Central Jakarta, Indonesia

The study by Agaus et al. explores changes in nutritional parameters, including body mass index (BMI) and serum albumin levels, in 175 patients undergoing intensive-phase therapy for tuberculosis (TB). The results show a significant increase in both BMI and serum albumin levels after completion of intensive treatment. The manuscript is well written, and the research questions are adequately addressed. However, several points require clarification.

 1. In the abstract, it is stated that post-treatment BMI measurements were conducted after 2 months of intensive therapy, whereas the Methods section indicates 2 weeks. Please clarify the correct time point and justify the selected period.

 2. Please specify the definition of the intensive phase of TB treatment in the study design, including the medications used.

 3. TCM is cited as a molecular rapid test; however, this is not a universally recognized abbreviation. Please use “molecular rapid test (MRT)” if referring to the same method, or define TCM clearly at first mention.

 4. While the increase in BMI and albumin levels is an important indicator of nutritional status and may suggest treatment success during the intensive phase, an important piece of information is missing: the conversion status of TB infection following intensive-phase treatment. Including this would strengthen the study by demonstrating whether improvements in BMI and albumin levels are consistent with treatment outcomes.

 5. In the Results section, the statement “After completion of the intensive phase of TB treatment, an increase in the proportion of patients with normal nutritional status was observed, rising to 52.6%, while the proportion of underweight patients decreased to 32%” requires clarification. Please specify the baseline percentages (i.e., the increase from …% to 52.6% and the decrease from …% to 32%).

 6. The authors cite Kumar et al. as supporting evidence for an increase in BMI after the intensive phase. However, Kumar et al. only reported differences in BMI between adverse drug reaction (ADR) and non-ADR groups, not changes before and after treatment. Please revise this interpretation.

 7. Please elaborate further in the Discussion on the variation in BMI responses depending on drug tolerance (as reported by Kalva et al.), as this provides important insight into treatment responses.

 8. Sontakke et al. is not listed in the reference list. Please revise accordingly.

 9. Please remove all publication years from the manuscript text, as references are already cited using a numeric system.

Is the work clearly and accurately presented and does it cite the current literature?

Yes

If applicable, is the statistical analysis and its interpretation appropriate?

I cannot comment. A qualified statistician is required.

Are all the source data underlying the results available to ensure full reproducibility?

Yes

Is the study design appropriate and is the work technically sound?

Yes

Are the conclusions drawn adequately supported by the results?

Partly

Are sufficient details of methods and analysis provided to allow replication by others?

Yes

Reviewer Expertise:

Molecular biomedicine

I confirm that I have read this submission and believe that I have an appropriate level of expertise to confirm that it is of an acceptable scientific standard.
